# β-Catenin Enhances Odontoblastic Differentiation of Dental Pulp Cells through Activation of Runx2

**DOI:** 10.1371/journal.pone.0088890

**Published:** 2014-02-10

**Authors:** Nana Han, Yong Zheng, Ran Li, Xianyu Li, Mi Zhou, Yun Niu, Qi Zhang

**Affiliations:** 1 State Key Laboratory Breeding Base of Basic Science of Stomatology (Hubei-MOST) and Key Lab of Oral Biomedicine Ministry of Education, School and Hospital of Stomatology, Wuhan University, Wuhan, Hubei, China; 2 Laboratory of Oral Biomedical Science and Translational Medicine, Department of Endodontics, School of Stomatology, Tongji University, Shanghai, China; 3 Department of Anatomy and Embryology, School of Medicine, Wuhan University, Wuhan, Hubei, China; University of South Florida College of Medicine, United States of America

## Abstract

An intense stimulus can cause death of odontoblasts and initiate odontoblastic differentiation of stem/progenitor cell populations of dental pulp cells (DPCs), which is followed by reparative dentin formation. However, the mechanism of odontoblastic differentiation during reparative dentin formation remains unclear. This study was to determine the role of β-catenin, a key player in tooth development, in reparative dentin formation, especially in odontoblastic differentiation. We found that β-catenin was expressed in odontoblast-like cells and DPCs beneath the perforation site during reparative dentin formation after direct pulp capping. The expression of β-catenin was also significantly upregulated during odontoblastic differentiation of *in vitro* cultured DPCs. The expression pattern of runt-related transcription factor 2 (Runx2) was similar to that of β-catenin. Immunofluorescence staining indicated that Runx2 was also expressed in β-catenin–positive odontoblast-like cells and DPCs during reparative dentin formation. Knockdown of β-catenin disrupted odontoblastic differentiation, which was accompanied by a reduction in β-catenin binding to the Runx2 promoter and diminished expression of Runx2. In contrast, lithium chloride (LiCl) induced accumulation of β-catenin produced the opposite effect to that caused by β-catenin knockdown. In conclusion, it was reported in this study for the first time that β-catenin can enhance the odontoblastic differentiation of DPCs through activation of Runx2, which might be the mechanism involved in odontoblastic differentiation during reparative dentin formation.

## Introduction

Dental pulps have regenerative capacity to form reparative dentin in cases of tooth injury [1]. Intense stimuli, such as cavity preparation and advanced dental caries, may causes death of odontoblasts and stimulate odontoblastic differentiation of the stem/progenitor cell populations of dental pulp cells (DPCs), which replace the necrotic odontoblasts; this is followed by reparative dentin formation [2], [3]. The odontoblastic differentiation of DPCs in response to tooth injury is essential to the reparative dentinogenesis of DPCs [4]. Previous studies suggested that dentin-like structures lined with odontoblast-like cells could be generated by isolated DPCs [5], [6]. Therefore, delineation the mechanism of odontoblastic differentiation of DPCs will be helpful for developing more biologically based strategies to treat dental tissue injury in clinics.

A number of molecular mechanisms are involved in odontoblastic differentiation of DPCs [4], [7], [8]. Among those is Wnt/β-catenin regulatory signaling pathways. Wnt/β-catenin plays crucial roles in the development of many self-renewing organs such as bone, gut, and skin and is required for the maintenance of homeostasis in these organs [9], [10]. β-catenin, as the central component of the Wnt/β-catenin pathway, is the bottleneck through which all signals pass [9]. Especially, β-catenin has also been found to have a central role in tooth development [11], [12]. For instance, inactivation of β-catenin in mesenchyme of developing tooth results in arrested tooth developmental at the bud stage, while forced β-catenin activation in embryogenesis or post-natal life causes ectopic tooth formation [13], [14]. There are studies also showing that tooth development and dental repair share some common molecular mechanisms [5], [15]. Additionally, robust studies have demonstrated that osteoblast differentiation, chondrocyte differentiation and adipocyte differentiation of stem/progenitor cells can be regulated by β-catenin [16], [17]. Given these previous findings, we hypothesize that β-catenin may participate in odontoblastic differentiation during reparative dentin formation.

β-catenin regulates a number of genes in various biological processes [18]–[20]. Among those, Runx2 is a transcriptional factor and known master regulator in controlling osteoblast and odontoblast differentiation [21]. It has been shown that expression of genes that are required for osteoblastic or odontoblastic differentiation is regulated by Runx2 [22]–[24]. For instance, Runx2 activates the transcription of the DSPP gene, which encodes two major dentin specific proteins DSP and DPP [22], [24]. Additionally, we and others have shown that Runx2 was upregulated during odontoblastic differentiation [25]–[27]. Although the important role of Runx2 in osteoblastic and odontoblastic differentiation has been well appreciated, how Runx2 itself in these processes is regulated remains unclear. Because β-catenin binds the Runx2 promoter and control its transcription [28], in this study, we aim to define the role of β-catenin in odontoblastic differentiation during reparative dentin formation and determine if such a role is fulfilled through activation of Runx2 by β-catenin.

## Materials and Methods

### Ethics statement

All animal experimental procedures were approved by the Institutional Animal Care Committee of Wuhan University. The study was approved by the Ethics Committee of Wuhan University School of Stomatology. Informed written consent was obtained from the parents/guardians of the children. Written informed assent was additionally obtained from the children who participated in the study.

### Tooth and tissue preparation

Direct pulp capping was prepared as previously described by us [29]. Briefly, 24 male Wistar rats (9 weeks old, weighing 200–250 g) were intraperitoneally anaesthetized with 20% (w/v) urethane (5 ml/kg). The maxillary teeth were cleaned and disinfected with 75% ethanol, and class V cavities with 1 mm diameter were prepared on the mesial surfaces of the maxillary first molars using #1/4 round burs. The cavities were then slightly perforated with the tip of a #8 sterile stainless-steel file. The bleeding was slight and stopped in several seconds by the pressure of a sterile cotton pellet. Excessively bleeding subjects were excluded from the study. Mineral trioxide aggregate (MTA) was placed on the perforation sites and then the cavity was sealed with glass ionomer cement (Fuji IX; GC, Tokyo, Japan). Eight rats of each group were sacrificed on day 0, 7 and 14 after tooth preparation. The maxillary first and second molars were dissected immediately after sacrifice and then fixed with 4% paraformaldehyde at 4°C for 24 h. The samples were then demineralized with 10% EDTA, dehydrated with gradient alcohols, and embedded in paraffin. Sagittal sections (5 µm) were obtained for hematoxylin-eosin (H&E) staining, immunohistochemical staining and immunofluorescence staining.

### Immunohistochemical staining and double immunofluorescence staining

After deparaffinization and rehydration, the sections were treated with pepsin (Zhongshan, Beijing, China) for 10 min. For immunohistochemical staining, endogenous peroxidase was blocked with 3% hydrogen peroxide followed by blocking with goat serum for 30 minutes. The sections were incubated with rabbit anti-β-catenin (Cell Signaling Technology, Beverly, MA, USA) and mouse anti-Runx2 (Abcam, Cambridge, UK) primary antibodies at 4°C overnight. The sections were washed and stained using the SP kit (Maixin-bio, Fuzhou, China) according to the manufacturer's instructions. Then stainings were visualized by diaminobenzidine solution (Maixin-bio, Fuzhou, China). For double immunofluorescence staining, the sections were incubated with primary antibodies mixtures of rabbit anti-β-catenin and mouse anti-Runx2 antibodies at 4°C overnight, followed by incubation with a mixture of goat anti-rabbit-Cy3 and goat anti-mouse-Dylight 488 antibodies for 1 h at room temperature. Sections were counterstained with DAPI to reveal nuclei. Photos were obtained by a fluorescent microscope with a camera (Leica, Wetzlar, Germany). Excitation and emission wavelengths were 405 and 425–460 nm (blue) for DAPI, 473 and 485–545 nm (green) for DyLight 488, 559 and 575–620 nm (red) for Cy3. Negative controls were obtained by substitution of primary antibodies with phosphate-buffered saline (PBS).

### Cell culture

Healthy premolars were collected from 13-to 15-year old patients undergoing premolar extractions for orthodontic reasons. Immediately after extraction, the teeth were washed with PBS and then fractured mechanically. The pulp tissue was removed with forceps and minced into small fragments (1–2 mm^2^). These explants were transferred to T-25 flasks containing alpha-modified Eagle's medium supplemented with 10% fetal bovine serum (Hyclone, Logan, UT, USA), 100 U/ml penicillin, and 100 µg/ml streptomycin at 37°C in a humidified atmosphere containing 5% CO_2_. After 2 weeks, the explants were discarded and DPCs were serially passaged. Cells at passage 3 were used in the study. The culture medium was changed at 3-day intervals.

Odontoblastic differentiation induction was performed as previously described [30]. Briefly, the DPCs were cultured in odontoblastic differentiaition medium containing alpha-modified Eagle's medium supplemented with 10% fetal bovine serum, antibiotics, 10 mM sodium β-glycerophosphate (Sigma-Aldrich, St. Louis, MO, USA), 10 nM dexamethasone (Sigma-Aldrich, St. Louis, MO, USA), and 50 µg/ml ascorbic acid.

### Lentivirus infection and LiCl treatment

β-catenin knockdown by shRNA was performed in the DPCs. A self-inactivating lentivirus containing a CMV-driven, enhanced green fluorescent protein (EGFP) reporter and a U6-driven shRNA against β-catenin (shRNA-β-cat sequence: 5-AAGUCCUGUAUGAGUGGGAAC-3) [31], [32] or nontargeting control (shRNA-NT sequence: 5-TTCTCCGAACGTGTCACGT-3) (GenePharma, Shanghai, China) was constructed by GenePharma as previously described. [33]. Conditions for lentivirus infection were optimized to ensure target-specific efficiency. The titer of virus used for the infection was 10^9^ pfu/ml. DPCs were seeded at a density of 1.5×10^4^ cells/cm^2^. The next day, the cells were infected for 24 h with supernatants containing viral particles (150 multiplicity of infection) in culture medium containing 5 µg/ml polybrene. The cells were then washed with PBS and treated with odontoblastic differentiation medium as mentioned above [34]. Accumulation of β-catenin was induced using LiCl, which specifically blocks GSK3 kinase activity [35]. The DPCs were cultured with either 10 mM LiCl or 10 mM sodium chloride (NaCl) as a control in odontoblastic differentiation medium [36], [37].

### Alizarin red staining

To demonstrate odontoblastic differentiation of the DPCs, the mineral deposits in the cultured cells were stained with alizarin red. After three PBS washes and fixation in 4% paraformaldehyde, the cells were incubated in 0.2% alizarin red (Sigma-Aldrich, St. Louis, MO, USA) solution for 20 min and washed twice in PBS.

### Alkaline phosphatase activity assay

Alkaline phosphatase (ALP) activity assay was performed using a commercial ALP activity assay kit (Nanjing Jiancheng Bioengineering Institute, Nanjing, China) according to the manufacturer's instructions. All results were normalized to total protein content. ALP activity was calculated as moles of p-nitrophenol/mg protein.

### Quantitative real-time PCR analysis

Total RNA was extracted from the cells with Trizol reagent (Invitrogen, Carlsbad, CA). Reverse transcription was carried out using a PrimeScript RT reagent kit (Takara Bio, Shiga, Japan) according to the manufacturer's instructions. Quantitative real-time PCR (qPCR) was performed using SuperReal Premix (Tiangen Biotech, Beijing, China) on the ABI 7500 Real-Time PCR System (Applied Biosystems, Foster City, CA, USA). Relative gene expression was calculated by the 2^−ΔΔCT^ method, and the values were normalized to the levels of GAPDH. Each experiment was performed in triplicate. Specific primers used were β-catenin,5-GTGCTATCTGTCTGCTCTAGTA-3(Forward),5-CTTCCTGTTTAGTTGCAGCATC-3(Reverse);Runx2,5-GAACCACAAGTGCGGTGCAA-3(Forward),5-ACTGCTTGCAGCCTTAAATGACTCT-3(Reverse); BSP,5-CAAGCATGCCTACTTTTATCCTC-3(Forward),5-CTTCTTGGGAAGCTGGATTG-3(Reverse);OCN,5-GGTGCAGCCTTTGTGTCCAA-3(Forward),5-CCTGAAAGCCGATGTGGTCA-3(Reverse);DSPP,5-CCATTCCCACTAGGACTCCCA-3(Forward),5-TGGCGATGCAGGTCACAAT-3(Reverse);DMP-1,5-AGGAAGTCTCGCATCTCAGAG-3(Forward),5-TGGAGTTGCTGTTTTCTGTAGAG-3(Reverse);ALP,5-AACATCAGGGACATTGACGTG-3(Forward),5-GTATCTCGGTTTGAAGCTCTTCC-3(Reverse);and GAPDH, 5-GCACCGTCAAGGCTGAGAAC-3(Forward),5-ATGGTGGTGAAGACGCCAGT-3(Reverse).

### Western blot analysis

Cultured DPCs were lysed in radioimmunoprecipitation (RIPA) buffer. Total proteins were measured using the BCA Protein Assay kit (Pierce Biotechnology, Rockford, IL, USA). Equal amount of protein was separated in 10% SDS-polyacrylamide gels and electrophoretically transferred onto a polyvinylidene difluoride transfer membrane. After blocking with 5% nonfat milk, the membranes were incubated overnight with specific primary antibodies against β-catenin (Cell Signaling Technology, Beverly, MA, USA), Runx2 (Abcam, Cambridge, UK), DSP (Santa Cruz Biotechnology, Inc, Santa Cruz, CA, USA), BSP (Beijing Biosynthesis Biotechnology, Beijing, China), or GAPDH (ProMab Biotechnologies, Richmond, CA, USA), followed by incubation with horseradish peroxidase(HRP)-conjugated secondary antibody (Antgene, Wuhan, China). The blots were then visualized by enhanced chemiluminescence (ECL).

### Chromatin immunoprecipitation (ChIP) assays

The changes in binding of β-catenin to the promoter of Runx2 were analyzed with ChIP assays as previously described [38]. After cross-linking with 1% formaldehyde and washing with PBS, the cells were collected and lysed. Cell lysates were sonicated to break chromatin into 200–1000 bp fragments. Sonicated samples were precleared with Protein A/G PLUS agarose beads (Santa Cruz Biotechnology, Inc, Santa Cruz, CA, USA) containing 1% salmon sperm DNA (Invitrogen, Carlsbad, CA, USA). 10% of the precleared samples were kept as inputs and the remaining samples were incubated overnight with anti-β-catenin or nonspecific IgG by rotation at 4°C. The immune complexes were precipitated with Protein A/G PLUS agarose beads and de-cross-linked. Precipitated DNA in the complexes was recovered by phenol extraction. The binding of β-catenin to the Runx2 promoter were quantified by qPCR amplification of the responsive elements located at the human Runx2 promoter. Primer sequences were: 5-CGTAGTAGTACACAACGCCG-3(Forward) and 5-GTTTCGTGTCTGT CTTCCCC-3(Reverse). qPCR reactions were carried out in the ABI 7500 Real-Time PCR System and enrichment of the immunoprecipitated DNA was normalized with the 1/10 inputs.

### Statistical analysis

Statistical significance was evaluated by the Student-Newman-Keuls multiple comparison test after a one-way ANOVA or Student's *t* test (comparing two groups) using software Sigma Plot 10.0 (Systat Software, Inc., Chicago, IL, USA), *P*<0.05 was considered to be statistically significant.

## Results

### Temporospatial expression of β-catenin during reparative dentin formation

**Figure 1:**
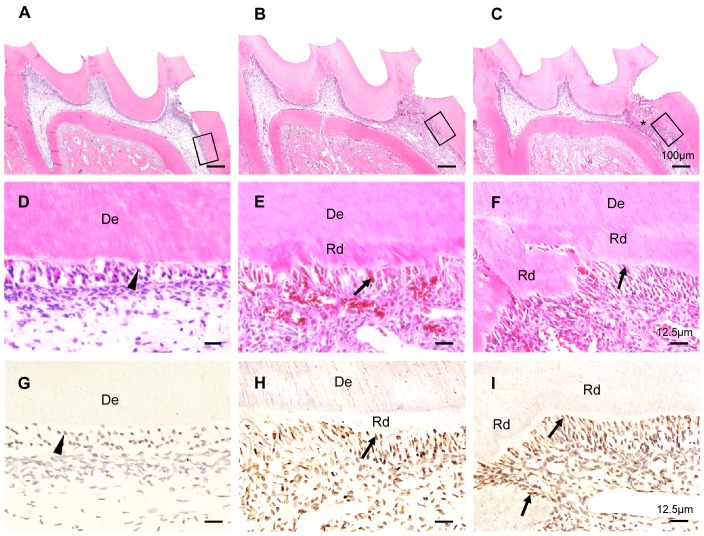
Temporospatial expression of β-catenin expression pattern during reparative dentin formation. H&E staining of maxillary first molar on day 0 (A and D), day 7 (B and E), day 14 (C and F) after tooth preparation. (A, B, C) The panorama of reparative dentin formation. (d) Odontoblasts (*arrowheads*) that lined the inner surface of dentin. (E and F) Columnar odontoblast-like cells (*arrows*) were lined along the inner surface of reparative dentin. The prepared molar presented a well-calcified reparative dentin bridge (*). Immunohistochemical staining of β-catenin on day 0 (G), day 7 (H), day 14 (I). β-catenin was strongly expressed in the odontoblast-like cells (*arrows*) and some dental pulp cells beneath the perforation, but was weakly expressed in the normal odontoblasts (*arrowheads*). De, dentin; Rd, reparative dentin. The photos are representative of three independent experiments.

To examine the temporospatial expression of β-catenin during reparative dentin formation, H&E and immunohistochemistry staining assays were performed on dental pulps at different stages of the reparative process. Immediately after tooth preparation (day 0), the odontoblast layer with weak β-catenin expression was detached from the dentin surface and the cells beneath the perforation site were irregularly arranged (Fig. 1A, D, and G). At 7 days post-preparation, reparative dentin was formed beneath the perforation site and a few odontoblast-like cells were surrounding the reparative dentin (Fig. 1B, E). At this stage, β-catenin was expressed not only in odontoblasts but also highly expressed in odontoblast-like cells and DPCs underneath the perforation (Fig. 1H). Two weeks after tooth preparation, there was formation of dentine bridge-like calcified tissue underneath the perforation site. We also found that some tall columnar odontoblast-like cells were lined along the inner surface of the calcified tissue, with some being entrapped in the calcified tissue (Fig. 1C, F). At this stage of reparation, β-catenin was found abundantly expressed in odontoblast-like cells and pulp cells beneath the perforation site (Fig. 1I). Additional, β-catenin was translocated into the nuclei of the odontoblast-like cells lining the reparative dentin (Fig. 1H, I). These data suggest that β-catenin participates in the odontoblastic differentiation of the DPCs during reparative dentin formation.

### Expression of β-catenin is upregualated during odontoblastic differentiation of DPCs

**Figure 2:**
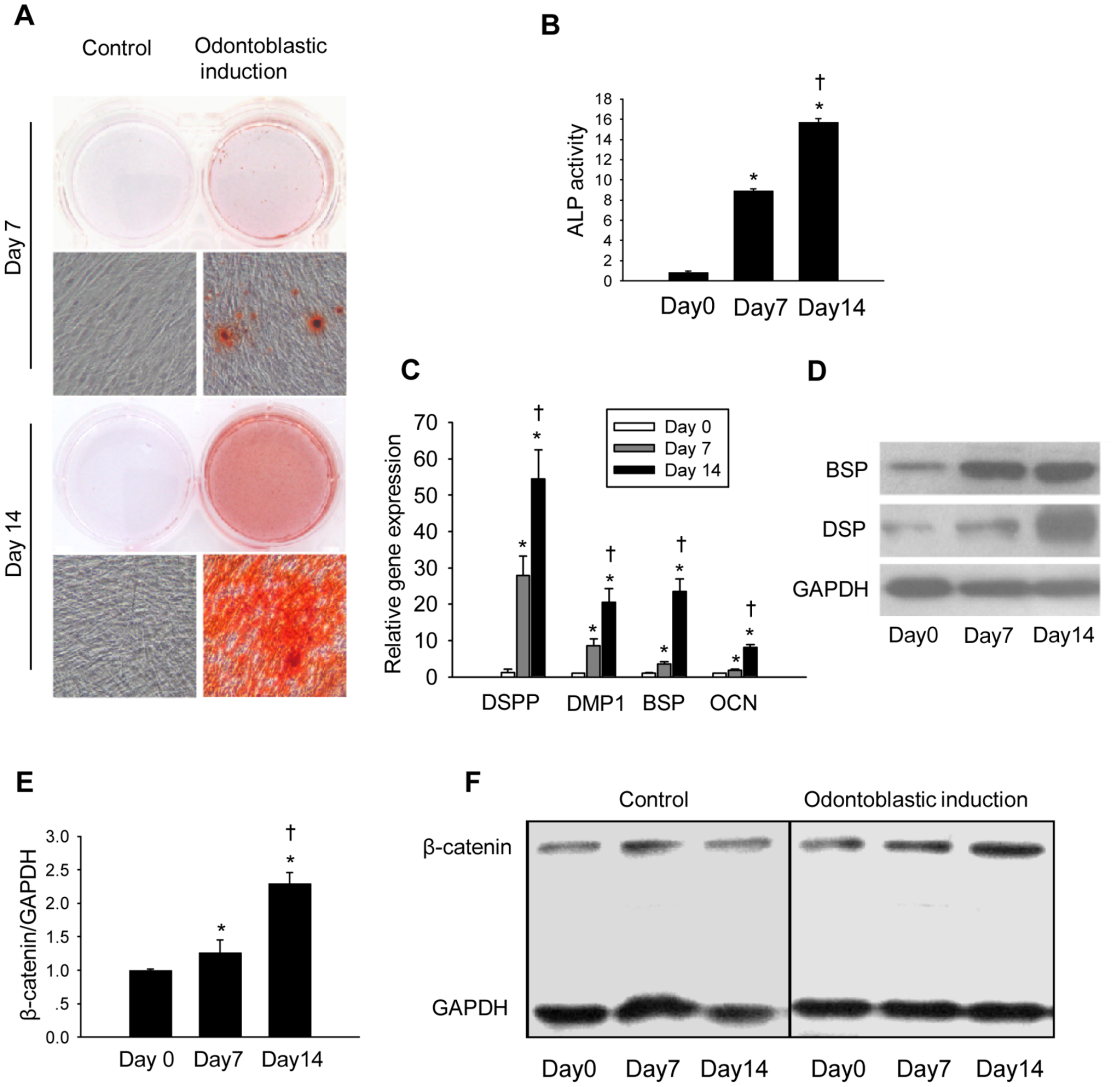
Expression of β-catenin is upregulated during odontoblastic differentiation of DPCs. (A) Alizarin red staining of DPCs on day 7 and 14 after odontoblastic induction. (B) ALP activity of DPCs on day 0, 7 and 14 after odontoblastic induction. ALP activity was calculated as moles of p-nitrophenol per mg protein. (C) mRNA levels of DSPP, DMP-1, BSP and OCN on day 0, 7, and 14 after odontoblastic induction. (D) Protein expression of DSP and BSP on day 0, 7, and 14 after odontoblastic induction. (E) mRNA level of β-catenin on day 0, 7 and 14 after odontoblastic induction. (F) Protein expression of β-catenin on day 0, 7 and 14 after odontoblastic induction. GAPDH served as an internal control. *P<0.05, compared with the previously adjacent cell group; †*P*<0.05, compared with cell group at day 0.

First, we confirmed odontoblastic differentiation of human DPCs by demonstrating mineralization nodules formation (Fig. 2A) and increased ALP activity (Fig. 2B) in differentiated cells. In the differentiated cells, the mRNA levels of odontoblastic differentiation-related genes including dentin sialophosphoprotein (DSPP), dentin matrix protein 1 (DMP-1), bone sialoprotein (BSP) and osteocalcin (OCN) were significantly upregulated (Fig. 2C). Consistently, protein levels of DSP and BSP were also increased after osteoblastic differentiation (Fig. 2D). The expression of β-catenin was examined at both mRNA (Fig. 2E) and protein levels (Fig. 2F). More importantly, we found that both mRNA and protein levels of β-catenin were greater on day 7 and day 14 after induction of differentiation than those in undifferentiated DPCs.

### Knockdown of β-catenin inhibits odontoblastic differentiation of DPCs

**Figure 3:**
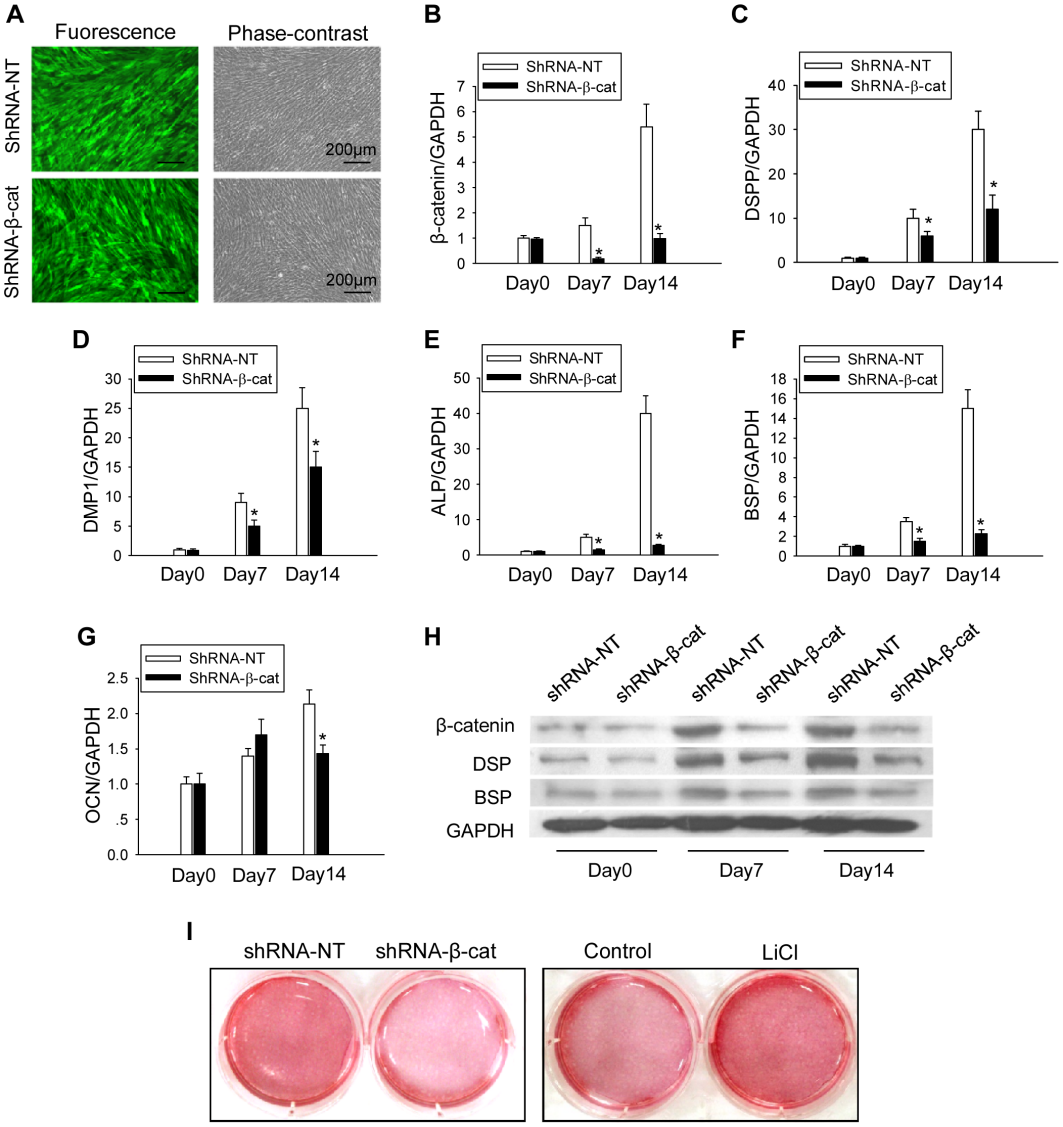
Knockdown of β-catenin inhibits odontoblastic differentiation of DPCs. (A) DPCs transfected with shRNA-NT or shRNA-β-cat on day 14 after transfection (EGFP positive). (B, C, D, E, F, G) Effects of shRNA-β-cat on the mRNA expression of β-catenin, DSPP, DMP1, ALP, BSP and OCN after odontoblastic induction, respectively. GAPDH served as an internal control. (H) Effect of shRNA-β-cat on the protein expressions of β-catenin, DSP and BSP on day 0, 7 and 14 after odontoblastic induction. (I) Alizarin red staining of DPCs on day 14 after odontoblastic induction with shRNA infection or LiCl treatment. *P<0.05, compared with previously adjacent cell group.

To further determine whether the upregulation of β-catenin was necessary for odontoblastic differentiation, we used lentivirus that express specific β-catenin shRNA to knock down β-catenin in DPCs and found that more than 90% of the infected cells were EGFP positive on day 14 after odontoblastic induction (Fig. 3A). qPCR and Western Blot analyses confirmed a marked decrease in β-catenin levels in shRNA-β-cat transfected cells compared with those transfected with shRNA-NT (Fig. 3B and H). In those cells with knockdown of β-catenin, we found a marked reduction in matrix mineralization and calcium nodule formation (Fig. 3I). The mRNA levels of DSPP, DMP-1, ALP and BSP were all significantly reduced in β-catenin knockdown cells on both day 7 and day 14 post induction, compared with those in cells infected with control lentivirus (Fig. 3B–F). Consistent with the mRNA expression, protein levels of DSP and BSP were also decreased in β-catenin knockdown cells (Fig. 3H). Although the mRNA level of OCN in the β-catenin knockdown cells was not significantly changed on day 7, it was reduced on day 14 after induction of odontoblastic differentiation (Fig. 3G).

### β-catenin accumulation induced by LiCl treatment promotes odontoblastic differentiation of DPCs

**Figure 4:**
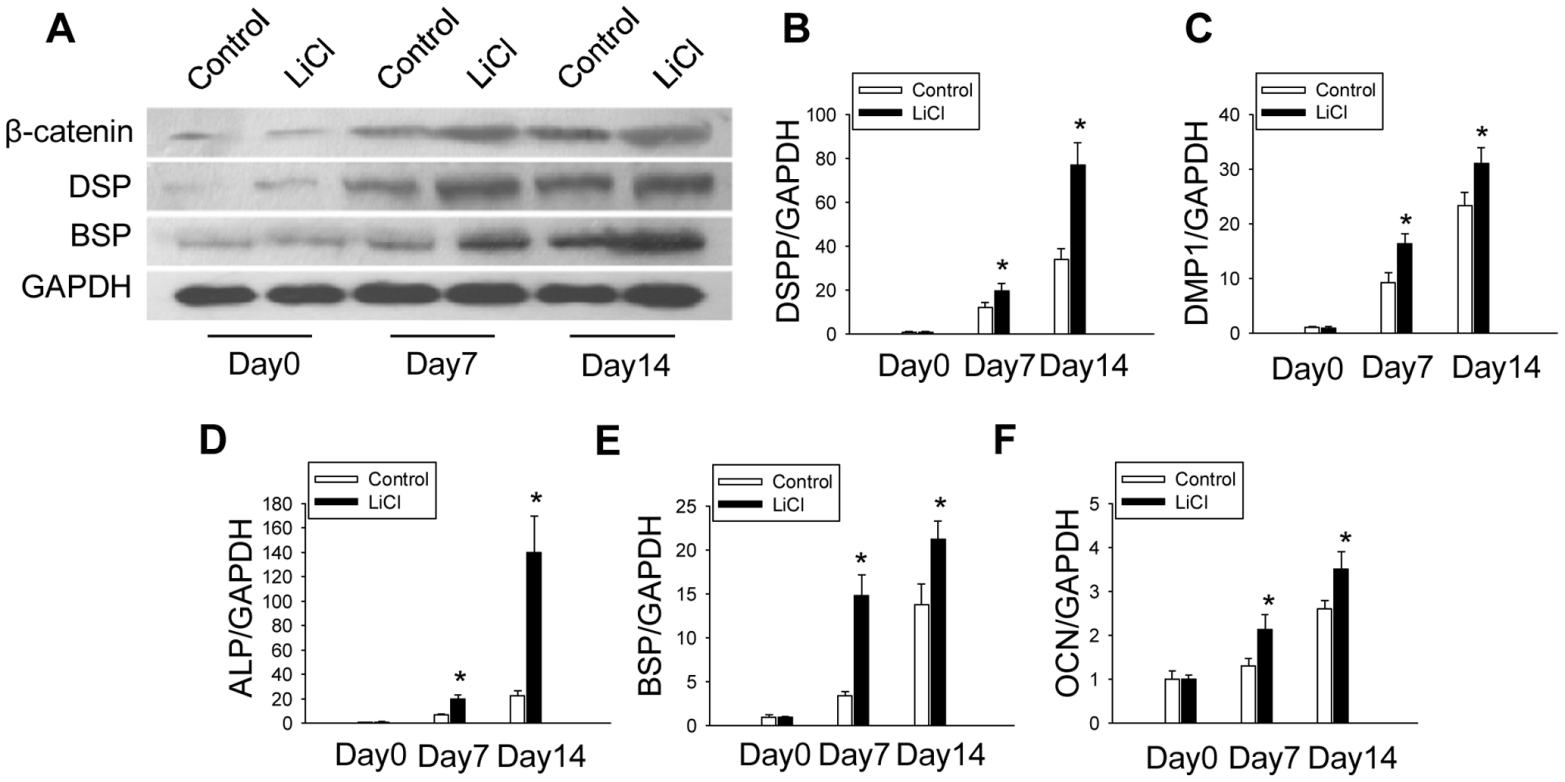
β-catenin accumulation induced by LiCl treatment promotes odontoblastic differentiation of DPCs. (A) Effects of LiCl treatment on protein expression of β-catenin, DSP and BSP. GAPDH served as an internal control. (B, C, D, E, F) mRNA levels of DSPP, DMP1, ALP, BSP and OCN after odontoblastic induction with LiCl treatment, respectively. *P<0.05, compared with previously adjacent cell group.

To further investigate the effect of β-catenin on odontoblastic differentiation of DPCs, we treated the cells with LiCl that was known to cause β-catenin accumulation [36], [37]. After odontoblastic differentiation induction with LiCl treatment for 14 days, the protein levels of β-catenin were higher in LiCl treated DPCs than those of the control groups (Fig. 4A). We found enhanced calcium nodule formation and matrix mineralization in LiCl treated cells (Fig. 3I). Additionally, mRNA levels of DSPP, DMP-1, ALP, BSP and OCN were upregulated in LiCl treated group on both day7 and day14 after induction of differentiation, compared with that of the control group (Fig. 4B–F). Consistent with the mRNA expression, protein levels of DSP and BSP were also enhanced on day 7 and day 14 in the LiCl treated group (Fig. 4A).

### β-catenin promotes odontoblastic differentiation through activation of Runx2

**Figure 5:**
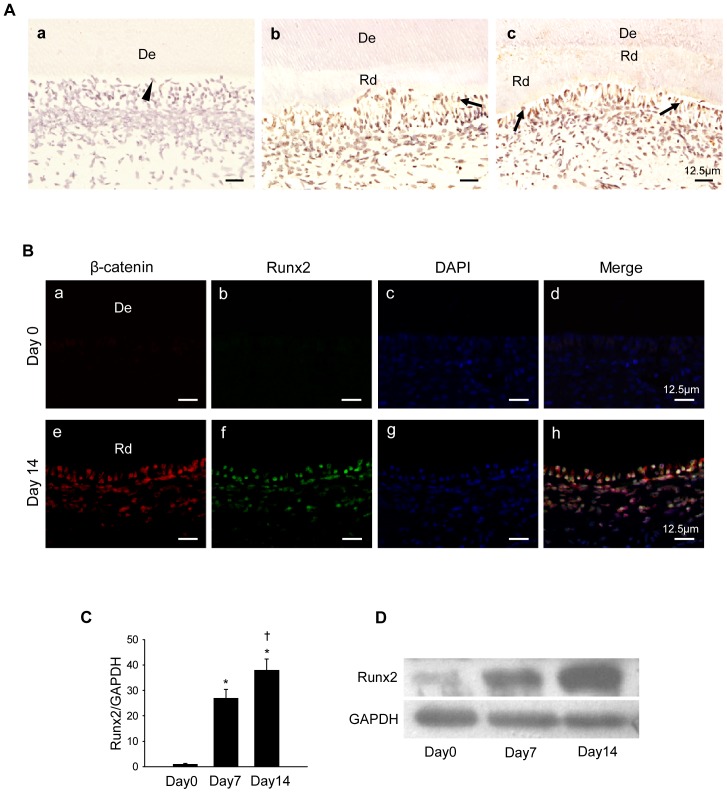
Expression of Runx2 during reparative dentin formation and odontoblastic differentiation of DPCs. (A) Immunohistochemical staining of runx2 on day 0 (a), day 7 (b), day 14 (c) after tooth preparation. *Arrowheads* indicate odontoblasts, and *arrows* indicate the odontoblast-like cells; De, dentin; Rd, reparative dentin. (B)Immunofluorescence staining for β-catenin (red) and Runx2 (green) on day 0 (a, b, c, d) and day 14 (e, f, g, h) after tooth preparation. Nuclei were counterstained by DAPI (blue). De, dentin; Rd, reparative dentin. (C) mRNA level of Runx2 on day 0, 7, and 14 after odontoblastic induction. (D) Protein expression of Runx2 on day 0, 7, and 14 after odontoblastic induction. GAPDH served as an internal control. *P<0.05, compared with the previously adjacent cell group; †*P*<0.05, compared with cell group at day 0.

**Figure 6:**
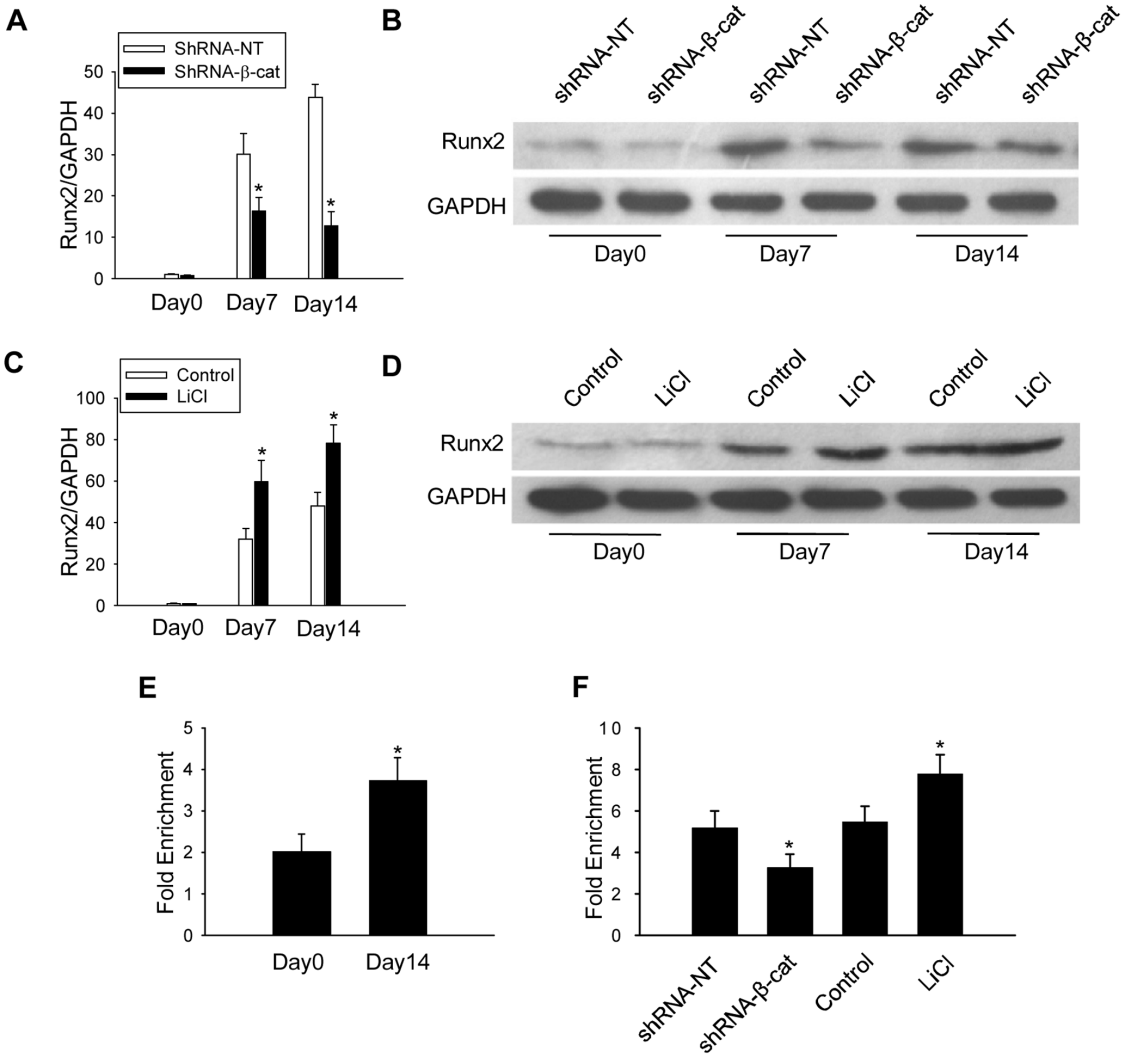
β-catenin promotes odontoblastic differentiation through activation of Runx2. mRNA level (A) and protein expression (B) of Runx2 in DPCs transfected with shRNA-NT or shRNA-β-cat. Effects of the activation of β-catenin by LiCl treatment on the expression of Runx2 at mRNA level (C) and protein expression (D). (E) Changes in binding of β-catenin to the chip region of promoter of Runx2 on day 0 and day 14 during odontoblastic differentiation induction. (F) Effects of the β-catenin knockdown and the accumulation of β-catenin by LiCl treatment on the binding of β-catenin to the promoter of Runx2. *P<0.05, compared with the previously adjacent cell group.

We found that Runx2 expression was undetectable in normal healthy dental pulps (Fig. 5A). However, on 7 and 14 days after tooth preparation, Runx2 expression was remarkably increased in the odontoblast-like cells and in some of the DPCs that surround the exposure sites, with the strongest staining located in the nuclei of the odontoblast-like cells (Fig. 5A). Immunofluorescence staining demonstrated that Runx2 was also expressed in many β-catenin–positive odontoblast-like cells and DPCs on day 14 after tooth preparation (Fig. 5B). qPCR and western blot results also found that Runx2 was significantly upregulated during odontoblastic differentiation of the DPCs (Fig. 5C, D). However, Runx2 expression in DPCs was greatly reduced when β-catenin was knocked down (Fig. 6A, B). Inversely, Runx2 expression was greatly upregulated by LiCl induced β-catenin accumulation (Fig. 6C, D). These findings suggest that Runx2 is a downstream mediator of β-catenin during odontoblastic differentiation. To test this hypothesis, we next determined whether β-catenin enhanced odontoblastic differentiation by binding to the promoter region of Runx2. As shown in Fig. 6E, odontoblastic differentiation led to an increased binding of β-catenin to promoter of Runx2 (Fig. 6E). However, knockdown of β-catenin decreased binding of β-catenin to the promoter of Runx2. In contrast, LiCl treatment increased the binding of β-catenin to the promoter of Runx2 on day 14 after induction of odontoblastic differentiation (Fig. 6F), consistent with β-catenin accumulation in LiCl treated cells.

## Discussion

Reparative dentin formation is a hallmark of dental pulp healing process following direct pulp capping with MTA, calcium hydroxide, and other materials [39]. MTA is a mechanical mixture of Portland cement, bismuth oxide and gypsum. When exposed to synthetic tissue fluids, Ca^2+^, that is released from MTA, reacts with phosphates to yield precipitates similar to hydroxyapatite [40]. Many studies showed that MTA is superior to conventional materials in promoting reparative dentin formation [41], [42]. Consistent with our previous study [29], there was progressive reparative dentin formation beneath the perforation site starting from day 7 after tooth preparation. More specifically, some columnar odontoblast-like cells that were newly differentiated from the stem/progenitor cell populations of DPCs were found surrounding or entrapped in the reparative dentin. Therefore, performing direct pulp capping with MTA is an ideal model to evaluate the pathophysiological process of dental pulp repair.

Previous studies suggest that there is a hierarchy of stem/progenitor cells in the adult dental pulp, including a small population of stem cells and relatively more committed progenitor cells [2]. Moreover, it is believed that dentin regeneration after tooth injury is mediated not by a single stem cell population but by many different populations in the dental pulp [43], [44]. Thus, we used DPCs to study the mechanism of odontoblastic differentiation during reparative dentin formation. In our study, the odontoblastic differentiation of DPCs was confirmed by increased ALP activity, mineralization nodules formation and upregulated odontoblastic differentiation-related genes, such as DSPP, DMP-1, BSP, and OCN.

β-catenin has been reported to play fundamental roles in various biological processes [9], [10], [45]. In the present study, we found β-catenin positive odontoblast-like cells and DPCs beneath the perforation and upregulation of β-catenin during *in vitro* odontoblastic differentiation of DPCs. These data indicate that β-catenin participates in reparative dentin formation and might also be necessary for odontoblastic differentiation of DPCs. We found that knockdown of β-catenin reduced the calcium nodule formation and the expression of DSPP, DMP-1, ALP, and BSP. In contrast, LiCl induced accumulation of β-catenin promoted calcium nodule formation accompanied by upregulated expression of DSPP, DMP-1, ALP, BSP and OCN. These findings suggest that odontoblastic differentiation of DPCs can be enhanced by β-catenin. These findings are consistent with a previous study showing that activation of β-catenin enhances odontoblastic differentiation of stem cells from the apical papilla [37]. However, Scheller *et al.* reported that stable expression of β-catenin inhibits differentiation and mineralization of dental pulp stem cells [46]. This discrepancy might be resulted from differences in the source of cells. There is more evidence to this controversy. Chen *et al.* found that activation of β-catenin signaling inhibited the differentiation of bone mesenchymal stem cells. However in cells that are committed to the osteoblast lineage, β-catenin enhanced osteoblast differentiation [47], [48]. Of note, DPCs used in this study contain not only a single stem cell population but also more differently committed progenitor cells. Interestingly, as a late-stage marker of odontoblastic differentiation [49], [50], OCN expression was not significantly changed by the knockdown of shRNA-β-cat at day 7. This seemingly inconsistent result might be explained by the fact that endogenous β-catenin might have little effect on the expression of OCN at the early stage of odontoblastic differentiation.

Previous studies showed that β-catenin interacts with several transcriptional co-factors to form DNA binding complex that regulates transcription of target genes. Miyabayashi *et al.* demonstrated that β-catenin/CBP complex plays a critical role in the maintenance of mES cell self-renewal capacity [51]. β-catenin also interacts with the TCF family, acting as a switch to determine differentiation-related gene transcription [52]. For example, Gaur *et al.* have shown that the β-catenin/TCF1 complex occupies the proximal promoter to control physiological levels of Runx2 [28]. The expression of osteogenesis or odontogenesis-related genes, such as Col1a1, Col1a2, osteopontin, BSP, OCN, fibronectin, MMP13, OPG, and DSPP, can be upregulated by Runx2 [22]–[24]. The effect of Runx2 during odontoblastic differentiation is dependent on the differentiation state of the target cells [21], [22]. In our study, we demonstrated that Runx2 was highly expressed in the odontoblast-like cells and pulp cells beneath the perforation in the process of reparative dentin formation. Furthermore, immunofluorescence staining indicated that Runx2 was coexpressed with β-catenin in the odontoblast-like cells and DPCs beneath the perforation site during reparative dentin formation. *In vitro* study of the odontoblastic differentiation of DPCs showed that expression of Runx2 was upregulated as well. Furthermore, we found that knockdown of β-catenin significantly diminished enhanced expression of Runx2 in differentiated DPCs, while LiCl induced β-catenin accumulation upregulated Runx2 expression. These findings indicate that Runx2 is a downstream mediator of β-catenin to promote odontoblastic differentiation of DPCs during reparative dentin formation. ChIP assays have consistently demonstrated that odontoblastic differentiation resulted in enhancement of β-catenin binding to the promoter region of the Runx2 gene. Additionally, knockdown of β-catenin caused a disassociation of β-catenin from the examined ChIP region, and accumulation of β-catenin by LiCl treatment enhanced the binding of β-catenin.

Collectively, our study showed that β-catenin was expressed in the DPCs and odontoblast-like cells beneath the perforation during reparative dentin formation. β-catenin enhanced odontoblastic differentiation of the DPCs *in vitro*. Additionally, odontoblastic differentiation of DPCs was accompanied by a remarkable increase of Runx2, which was controlled by β-catenin. Moreover, coexpression of Runx2 and β-catenin was observed in the odontoblast-like cells and DPCs beneath the perforation site during reparative dentin formation. Therefore, the β-catenin/Runx2 pathway may enhance odontoblastic differentiation of DPCs during reparative dentin formation. It has been reported that Runx2 can regulate the transcription of the DSPP gene [22], [24], which encodes two major dentin specific proteins DSP and DPP. Other genes targeted by β-catenin/Runx2 during odontoblast differentiation remain to be identified in our future studies. Although we found that β-catenin regulates Runx2 expression during odontoblastic differentiation, it is still unclear if Runx2 directly mediates the promotive role of β-catenin in this process. Therefore, further studies are warranted to delineate the network of β-catenin regulatory mechanism in odontoblastic differentiation of DPCs during reparative dentin formation.
